# RESOLFT nanoscopy with photoswitchable organic fluorophores

**DOI:** 10.1038/srep17804

**Published:** 2015-12-07

**Authors:** Jiwoong Kwon, Jihee Hwang, Jaewan Park, Gi Rim Han, Kyu Young Han, Seong Keun Kim

**Affiliations:** 1 Department of Biophysics and Chemical Biology, Seoul National University, Seoul 08826, Korea; 2 Department of Chemistry, Seoul National University, Seoul 08826, Korea; 3 Howard Hughes Medical Institute, Urbana, Illinois 61801, USA; 4 Department of Physics and Center for the Physics of Living Cells, University of Illinois at Urbana-Champaign, Urbana, Illinois 61801, USA

## Abstract

Far-field optical nanoscopy has been widely used to image small objects with sub-diffraction-limit spatial resolution. Particularly, reversible saturable optical fluorescence transition (RESOLFT) nanoscopy with photoswitchable fluorescent proteins is a powerful method for super-resolution imaging of living cells with low light intensity. Here we demonstrate for the first time the implementation of RESOLFT nanoscopy for a biological system using organic fluorophores, which are smaller in size and easier to be chemically modified. With a covalently-linked dye pair of Cy3 and Alexa647 to label subcellular structures in fixed cells and by optimizing the imaging buffer and optical parameters, our RESOLFT nanoscopy achieved a spatial resolution of ~74 nm in the focal plane. This method provides a powerful alternative for low light intensity RESOLFT nanoscopy, which enables biological imaging with small organic probes at nanoscale resolution.

Since 1994, a number of far-field optical microscopy techniques have been proposed to overcome the optical diffraction limit^1^,^2^,^3^. Although each of these fluorescence nanoscopy (“super-resolution imaging”) techniques uses distinct strategy, all of them share an identical core mechanism, i.e., switching between a ‘bright’ (fluorescent, “ON”) state and a ‘dark’ (non-fluorescent, “OFF”) state^4^. In contrast to the single-molecule based localization techniques^5^,^6^,^7^,^8^, the coordinate-targeted nanoscopy techniques such as stimulated emission depletion (STED) or ground state depletion (GSD) microscopy generally use the reduction of the effective focal volume. This is done by concentrically overlaying a circular excitation beam with a donut-shaped depletion beam, leading to the pre-emptive decay of off-center fluorophores to a dark state^9^,^10^. The singlet ground state often acts as the dark state in STED while the first triplet excited state plays the same role in GSD.

There is yet another approach, simply called RESOLFT (reversible saturable optical fluorescence transition), which usually exploits reversibly photoswitchable fluorescent proteins (RSFPs)^11^,^12^,^13^,^14^. Due to the fact that the photoinduced dark states are thermally stable^15^, RESOLFT nanoscopy requires a much lower laser intensity than STED and GSD microscopy. It also leads to a low residual fluorescence level. Because RSFPs can be easily expressed in living cells via genetic engineering, RESOLFT nanoscopy has been considered as a promising tool for biological applications of super-resolution imaging^16^,^17^,^18^. For example, thanks to the low operating laser intensity, even a living brain tissue was visualized with nanometric resolution for a long observation time with negligible photodamage^19^. This feature also allows parallelized RESOLFT nanoscopy with >20,000 doughnut-like intensity minima, which greatly reduces the acquisition time^20^. Furthermore, a recently developed RESOLFT technique enables a facile dual-color imaging of spectrally undistinguishable fluorescent proteins by using the difference of fluorescent kinetics^21^.

Compared to fluorescent proteins, RESOLFT nanoscopy with commercially available organic fluorophores remains challenging for a biological system although the RESOLFT technique itself has been demonstrated with a few organic dyes^22^,^23^. The main reason that organic dyes have rarely been used in RESOLFT nanoscopy is the poor resistance to the photobleaching over multiple switching cycles (see Supplementary Table S1 online). We here adopt the most widely used cyanine dyes to implement organic dye-based RESOLFT nanoscopy. When a cyanine dye such as Cy5 or Alexa647 is excited in a buffer solution containing a small primary thiol, it forms a long-lived non-fluorescent state as a result of the thiol addition to the polymethine chain of the cyanine dye^24^,^25^,^26^. Irradiation with a light of a wavelength shorter than 550 nm restores the photoproduct to the original fluorescent form^25^,^26^. Since it is well known that a neighboring fluorophore that absorbs the restoring light can greatly enhance the restoring efficiency^25^,^27^, we attempted to maximize the photoswitching and labeling efficiencies by covalently linking the ‘activator’ and ‘reporter’ dye pair, Cy3-Alexa647, into a ‘heterodimer’ (Fig. 1a)^27^,^28^. We then performed a series of pump-probe experiments to examine the photoswitching characteristics of this heterodimer under RESOLFT imaging conditions, and successfully obtained the RESOLFT images of nuclear pore complex, mitochondria and microtubule using organic fluorophores.

## Results & Discussion

### Optical properties of Cy3-Alexa647 heterodimer

**Figure 1:**
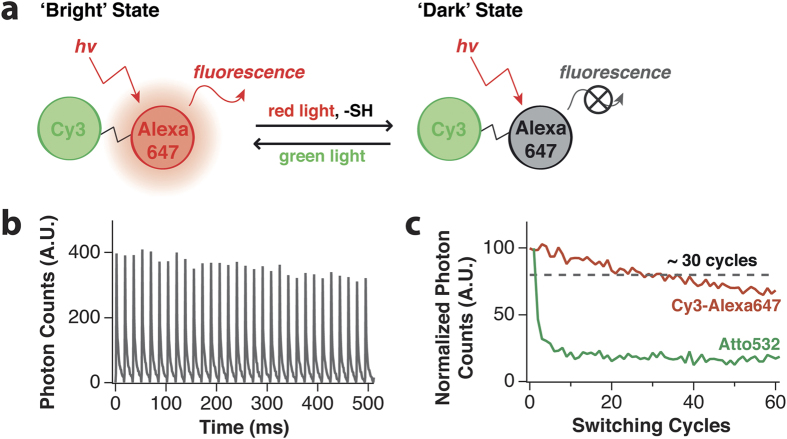
(**a**) Schematic illustration for the photoswitching of the Cy3-Alexa647 heterodimer. The ‘reporter’, Alexa647, emits the fluorescence upon a red laser illumination during which it forms a dark state, whereas the ‘activator’, Cy3, helps the activation process under green laser irradiation. (**b**) Fluorescence emission showing the on/off cycles upon repetitive irradiation under optimized illumination condition (activation: 532 nm, 0.5 ms, 1.6 kW/cm^2^; depletion: 633 nm, 15 ms, 4.0 kW/cm^2^) and imaging buffer (143 mM of MEA and 100 mM of KI). (**c**) Photobleaching behavior of Cy3-Alexa647 in imaging buffer and Atto532 in PVA.

In order to see if organic fluorophores can be used in RESOLFT nanoscopy, we prepared the Cy3-Alexa647 heterodimer as described in Methods^28^. It shows the absorption maximum at 549 and 652 nm (extinction coefficient: 53,200 and 63,300 M^−1^cm^−1^, respectively) and the emission maximum at 564 and 668 nm. In the heterodimer, the fluorescence lifetimes of Cy3 and Alexa647 were 0.39 and 0.97 ns, and the fluorescence quantum yield of Alexa647 was 0.15. The averaged efficiency of resonance energy transfer from Cy3 to Alexa647 was ~0.83. Next, we prepared the heterodimer labeled microtubules in primary Human-skin-fibroblast cells and examined its photoswitching capability over many repeated cycles under optimized imaging conditions that will be described below (Fig. 1b,c). The fluorescence intensity of the sample was measured while repetitively irradiating red (633 nm) and green (532 nm) lasers. A corresponding fluorescence on/off signal was obtained for more than dozens of times with a minor degree of photobleaching (Fig. 1b). More than 80% of the Cy3-Alexa647 heterodimers survive after ~30 switching cycles and thus appear to be far more fatigue-resistant against photobleaching than other organic dye Atto532 in poly(vinyl alcohol) (PVA) that used in GSD microscopy (Fig. 1c)^10^. The durability of the Cy3-Alexa647 photoswitching may ensure its applicability to the RESOLFT scheme.

### Experimental scheme for pump-probe measurements

In order to successfully implement organic fluorophores in RESOLFT nanoscopy, experimental conditions need to be optimized to improve the spatial resolution while avoiding photobleaching at the same time. The spatial resolution of RESOLFT nanoscopy is critically governed by the transition efficiency that is determined by two factors: the cross section of the optical transition and the photon flux of the depletion laser^11^. The former is highly affected by the chemical composition of the imaging buffer and the photophysical properties of the ‘reporter’ dye, whereas the latter is controlled by the irradiation intensity and the duration of the depletion laser. On the other hand, for fluorescence recovery, the cross section for photoactivation and the photon flux of the activation laser are the key factors.

**Figure 2:**
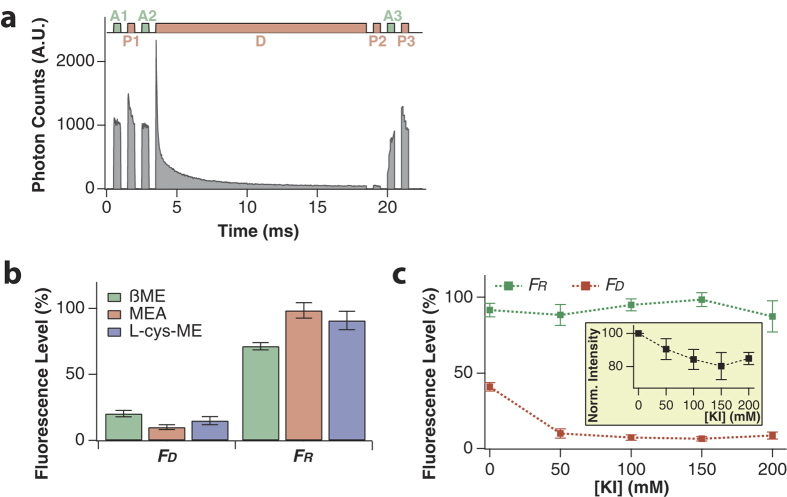
(**a**) Laser beam train sequence for the pump-probe experiment. One sequence of beams is applied to each pixel during sample scanning. (**b**) Effect of primary thiols (with 50 mM KI) on the switching characteristics of the Cy3-Alexa647 heterodimer. (**c**) The residual and recovered fluorescence levels against KI concentration in 143 mM MEA solution. Inset, the fluorescence intensity decreases with increasing concentration of KI. (Error bars: standard deviations).

We performed pump-probe experiments in the millisecond range with a home-built microscope so as to analyze the photoswitching characteristics of the heterodimer labeled to microtubules in the imaging buffer (Fig. 2a). A series of laser beams was repeatedly applied to a tightly focused region of the sample while it was being scanned. We combined seven beams in a period, which consists of three activation beams (532 nm; *A1*, *A2*, and *A3*), one depletion beam (658 nm; *D*), and three probe beams (633 nm; *P1*, *P2*, and *P3*). Beams *A1*, *A2*, and *A3* are applied when the sample is needed to be activated, a beam *D* actually depletes the fluorophore to the dark state, and beams *P1*, *P2*, and *P3* read out the fluorescence level after depletion (*P2*) and activation (*P3*) and also from reference state (*P1*). The switching efficiency was analyzed by calculating the residual fluorescence level 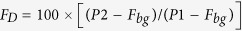  and the recovered fluorescence level 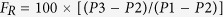, where *F*_*bg*_ represents the background signal measured on an area where there is no fluorophore. In order to obtain correct information about photoswitching, it is essential to prevent photobleaching and run many switching cycles to maximize the signal-to-noise (S/N) ratio. In order to satisfy these conditions, we scanned the immobilized heterodimers on a 8 × 8 μm^2^ area with a sufficiently large pixel size of ~600 nm, which is certainly larger than the diffraction limit. On each pixel, we recorded time-dependent fluorescence intensity with time resolution of 22 μs over 22.5 ms, where the laser sequence was applied only once to a pixel to ensure minimal photobleaching. All of the pump-probe measurements were carried out 5 times in separate regions and their values were averaged.

### Optimization of imaging buffer

Increasing the transition cross section was achieved by optimizing the imaging buffer. First, we investigated the switching efficiency of three primary thiols: β-mercaptoethanol (βME), β-mercaptoethylamine (MEA), and L-cysteine methyl ester (L-cys-ME). Since the thiols show a saturation behavior in the concentration range of >100 mM to form the dark state^26^, we used 143 mM of thiols that corresponds to 1% v/v of βME. Figure 2b compares the *F*_*D*_ and *F*_*R*_ values of our fluorophore in the presence of individual thiol species and oxygen scavengers with 50 mM KI. It is obvious that MEA gives the best results for both switching directions, and we thus used MEA in all pump-probe measurements and RESOLFT imaging.

Potassium iodide (KI) is another crucial element to improve the transition cross section, as the iodide increases the inter-system crossing rate by the heavy atom induced spin-orbit coupling. A previous study found that the photoinduced adduct formation can be accelerated upon increasing the iodide concentration^24^, suggesting that the reaction pathway involves the triplet state. The fact that KI decreases *F*_*D*_ substantially while keeping *F*_*R*_ at around 100% (Fig. 2c) indicates that it increases the transition cross section of the heterodimer but little affects both the photoactivation cross section and the photobleaching quantum yield. Unfortunately, KI also decreases the fluorescence intensity of the ‘bright’ state molecules due to a high population in the triplet state (Fig. 2c, inset). Considering all these effects on the fluorescence intensity and depletion rate, we chose a KI concentration of 100 mM for all our experiments.

### Determination of the optical imaging conditions

Following the optimization of the imaging buffer conditions, we tried to optimize the photon flux by controlling the depletion and activation beams. Since the beam duration and peak intensity vary inversely for a constant photon flux, we fixed the beam duration and changed the peak intensity to control the photon flux for experimental convenience. Because the beam duration is the most critical factor for the total image acquisition time, it is desirable to minimize the beam duration without causing significant photobleaching. Considering all these factors, we used depletion and activation beams of 15 and 0.5 ms duration, respectively.

**Figure 3:**
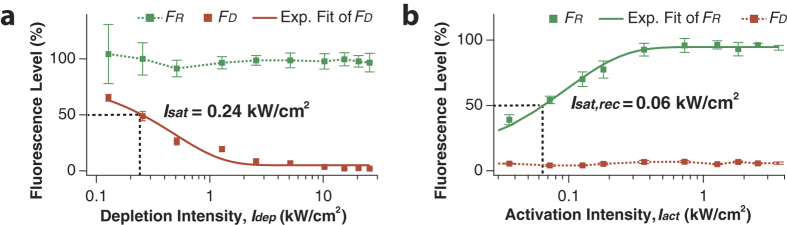
Light intensity dependence of the depletion ((**a**), red markers) and the activation, ((**b**), green markers) on fluorescence intensity. Exponential fittings (solid red and green lines) yield the saturation intensity of 0.24 kW/cm^2^ and 0.06 kW/cm^2^, respectively. (Error bars: standard deviations).

In order to investigate the effect of depletion intensity, *I*_*dep*_, on photoswitching, we measured *F*_*D*_ as we varied *I*_*dep*_ while keeping the activation intensity, *I*_*act*_, at a constant level (Fig. 3a). With only 2.5 kW/cm^2^ of *I*_*dep*_, *F*_*D*_ was depleted below 10%, which is much lower depletion intensity than used in STED but comparable to that used in GSD. Without photoactivation, the thermal lifetime of the dark state is of the order of hours^24^, which results in nearly complete depletion below 2% with higher *I*_*dep*_. Exponential fitting of the depletion curve yielded a saturation intensity, *I*_*sat*_, of 0.24 kW/cm^2^ (Fig. 3a, solid red line), which amounts to 0.55 μW and is a typical value of laser intensity needed to turn off half of the fluorescence. *I*_*sat*_ also can be used to estimate the size of the effective focal point, 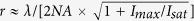, where *I*_*max*_ indicates the applied depletion intensity. Based on the above equation, it is readily estimated that our proposed experimental scheme should easily achieve >4-fold enhancement in lateral resolution with *I*_*dep*_ less than ~5 kW/cm^2^. In our tested range of *I*_*dep*_ (≤30 kW/cm^2^), *F*_*R*_ stayed at a constant level with little fluctuations (Fig. 3a, dotted green line), which indicates that the depleting photon flux neither affect the activation process nor induce significant photobleaching in this intensity range.

Optimization of *I*_*act*_ is also important to acquire high-resolution images because without perfect activation, a considerable amount of fluorescence signal would be lost due to the ‘dark’ fluorophores in the reduced focal volume. Moreover, excess activating photon flux tends to greatly facilitate photobleaching of the reporter through diverse pathways including the fluorescence resonance energy transfer^29^. Figure 3b shows that at *I*_*dep*_ of 5.1 kW/cm^2^, *F*_*R*_ increases with *I*_*act*_ in the lower range of intensity (<0.4 kW/cm^2^), suggesting that the activating photon flux was not saturated in this range. In the higher range of intensity (up to 4 kW/cm^2^ as tested), we see a nearly perfect photoactivation efficiency of greater than 95% without severe photobleaching. The result was well fitted by an exponential function that yielded a saturation intensity of 0.06 kW/cm^2^ for the activation process (Fig. 3b, solid green line). Again, *F*_*D*_ stayed constant at the initial level in the tested *I*_*act*_ range, indicating that the activating photon flux is completely decoupled from the depletion process (Fig. 3b, dotted red line). It has been known that it is possible to directly photoactivate Alexa647 (with no adjacent Cy3) in the dark state^25^; however, this approach required ~3 kW/cm^2^ of 532-nm laser with 5 ms of illumination time that causes severe photobleaching, whereas Cy3-Alexa647 required only 0.06 kW/cm^2^ of 532-nm laser with 0.5 ms of illumination time (~500-fold enhancement in photoactivation efficiency), leading to negligible photobleaching. From a simple set of coupled kinetic equations, we were able to obtain the kinetic rate constants of *k*_*dep*_ = 0.06 cm^2^W^−1^s^−1^ and *k*_*act*_ = 14.54 cm^2^W^−1^s^−1^ (Supplementary Fig. S1 online), which are in good agreement with the values obtained from previous work (*k*_*dep*_ ~ 0.015 cm^2^W^−1^s^−1^ in the absence of KI, with an extrapolated estimate of 0.06 ~ 0.07 cm^2^W^−1^s^−1^ at 100 mM KI; *k*_*act*_ ~ 12 cm^2^W^−1^s^−1^)^24^.

### RESOLFT imaging of Cy3-Alexa647 labeled subcellular structures in fixed cells

**Figure 4:**
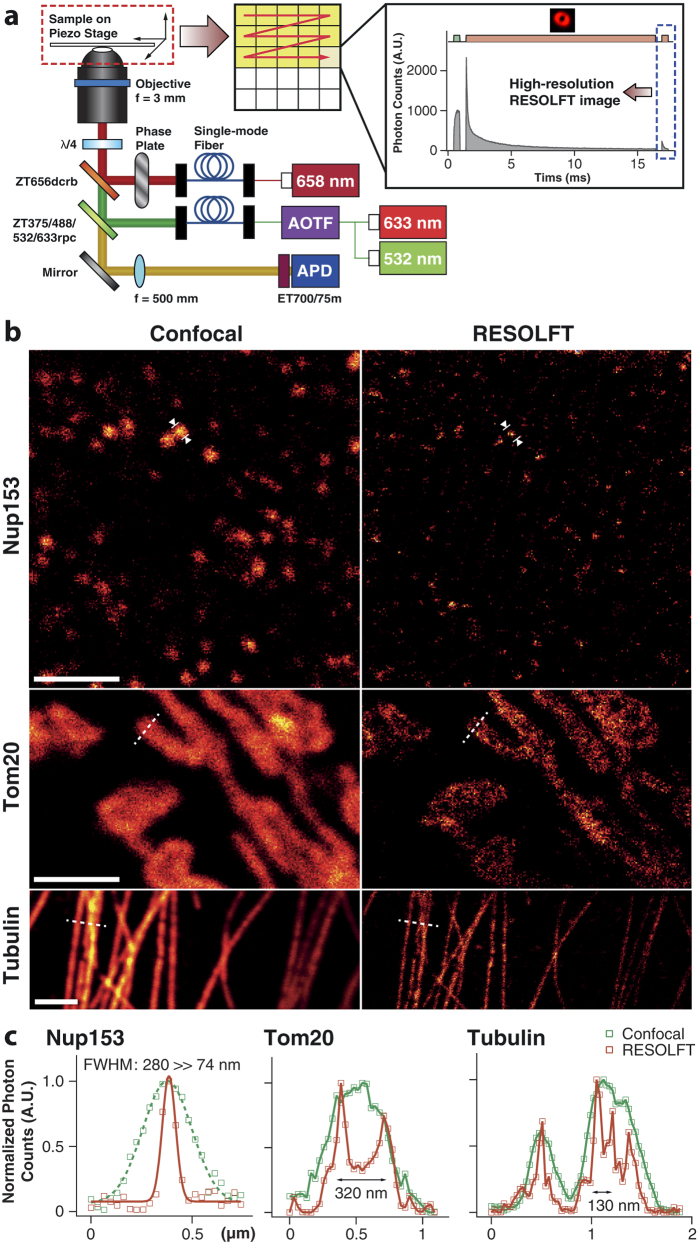
(**a**) RESOLFT nanoscopy setup and schematic illustration of RESOLFT imaging. Activation, depletion and probe beams are sequentially applied to every pixel during sample scanning (along the red arrow). The probe beam reads out the emitted photons from the reduced effective focal volume. (**b**) Confocal (left) and RESOLFT (right) images of subcellular structures in fixed cells stained with the Cy3-Alexa647 heterodimer. (Nup153: nuclear pore complex in NIH3T3 cells; Tom20: mitochondria in primary Human-skin-fibroblast cells; Tubulin: microtubules in primary Human-skin-fibroblast cells). All images are raw data. Scale bars: 2 μm. (**c**) Fluorescence line profiles along the white marked region (Nup153) and white dotted lines (Tom20 and Tubulin) in (**b**).

Finally, we demonstrated the feasibility of RESOLFT imaging of subcellular structures labeled by the covalently-linked Cy3-Alexa647 heterodimer in fixed cells. We applied three beams that are comprised of activation (0.5 ms, 1.6 kW/cm^2^), depletion (15 ms, 4.0 kW/cm^2^), and probe (0.5 ms, 2.1 kW/cm^2^) beams in a pump-probe manner with the probe beam used to visualize the RESOLFT image (Fig. 4a). The depletion beam was donut-shaped while the other two beams were of the circular type. We used the imaging buffer containing 143 mM of MEA and 100 mM of KI to ensure the lowest *F*_*D*_ of ~5% and *F*_*R*_ greater than 95% without severe photobleaching.

We obtained RESOLFT images of nuclear pore complex (Nup153) labeled with the Cy3-Alexa647 heterodimer in NIH3T3 cells (Fig. 4b). In contrast to a confocal image, the RESOLFT image showed much enhanced resolution. The experimental full-width at half maximum (FWHM) of our RESOLFT nanoscope obtained from eight random positions was 74 ± 20 nm (see Supplementary Fig. S2 online), which is about one fourth of that of a confocal microscope (279 ± 35 nm). A representative line profile is depicted in Fig. 4c. Additionally, we imaged mitochondria and microtubule in primary Human-skin-fibroblast cells targeted to the translocases of outer mitochondrial membranes (Tom20) and the major components of microtubules (Tubulin), respectively. Our RESOLFT technique visualized the hollow structure of mitochondria (Fig. 4b,c – Tom20) and clearly resolved adjacent filaments separated by up to ~130 nm in microtubules where confocal microscopy yielded only a blurred image of one thick fiber (Fig. 4b,c – Tubulin).

We note, however, that our resolution is not as high as can be expected from our pump-probe measurements due to a high extent of photobleaching. It was reported that Alexa647 is photobleached after ~50 switching cycles in aqueous buffer^30^, and even in our optimized imaging conditions, smaller pixel size (<32 nm) may induce rather severe bleaching due to the excessively large number of switching cycles. With more powerful reagents suppressing photobleaching, our method has a good prospect of offering even better spatial resolution with low operating power.

In conclusion, we successfully introduced the covalently-linked photoswitchable heterodimer Cy3-Alexa647 into RESOLFT nanoscopy in aqueous buffer. We optimized the imaging conditions by pump-probe fluorescence measurements and demonstrated the feasibility of our method by taking a super-resolution imaging of intracellular structures immunostained with the Cy3-Alexa647 heterodimer. This method will open a new, efficient way to achieve sub-diffraction-limit imaging with commonly used organic fluorophores and low intensity lights, which will greatly extend its applicability to a variety of biomolecules, for instance, DNA, RNA and lipids as well as proteins.

## Methods

### Experimental Setup

A home-built fluorescence confocal microscope was used to examine photoswitching characteristics in various conditions and to image the Cy3-Alexa647 labeled to biological samples. Three continuous-wave lasers (532 nm: Samba^TM^ 532, Cobolt; 633 nm: 25-LHP-928-230, CVI Melles-Griot; 658 nm: TECRL-50GC-658-TTL-A, World Star Tech) were employed to generate the activation, probe, and depletion beams, respectively. The outputs of the 532-nm and 633-nm lasers were combined by using a dichroic mirror (ZT532rdc, Chroma) and passed through an acousto-optic tunable filter (AOTFnC-VIS-TN, AA Optoelectronic) that performs active modulation of the millisecond beams. On the other hand, the 658-nm laser was modulated by an external TTL signal. All three laser beams were spatially filtered with single-mode fibers (Φ = 4.2 μm, P1-630PM-FC, Thorlabs) and finally combined in the same beam path using two dichroic mirrors (ZT656dcrb and ZT375/488/532/633rpc, Chroma). The combined beams were sent through an oil-immersion objective (UIS2 series, PlanApo N, NA1.45, 60x, Olympus). In RESOLFT imaging, a vortex phase plate (VPP-1, RPC Photonics) was inserted in the beam path of the 658 nm laser. Achromatic λ/4 and λ/2 retarders (RAC 3.4.15, RAC 3.2.15 and RAC 4.2.15, Bernhard Halle Nachfl.) were used to make circularly polarized lights to generate minimum intensity at the center of the donut beam and to yield uniform resolution in the focal plane^31^. The sample was placed on a piezo-stage (Max311, Thorlabs) that can be scanned with <5-nm accuracy. All electrical input signals were generated by two analog output boards (PCI-6731 and PCI-6713, National Instruments) to control the 658-nm laser, AOTF and piezo-stage. The fluorescence signal was filtered through an emission filter (ET700/75m, Chroma) and fed into a multi-mode fiber (Φ = 62.5 μm, M31L02, Thorlabs) that works as a pinhole. The fluorescence signal was collected by an avalanche photodiode (SPCM-AQR-14FC, Perkin Elmer) and processed by a multi-channel scaler (P7882, Fast ComTech) that converted the analog signal to photon counts, which were finally visualized by an imaging program, Imspector.

### Synthesis of the Cy3-Alexa647 heterodimer

The Cy3-Alexa647 heterodimer was synthesized by covalently linking the Cy3 bis-NHS ester (PA23000, GE Healthcare) and the Alexa647 cadaverine (MOP-A-30679, Invitrogen) in anhydrous dimethyl sulfoxide (276855, Sigma Aldrich) with 8 mM triethylamine (T0886, Sigma Aldrich)^27^,^28^ (see Supplementary Fig. S3 online). The molar ratio of the reactants was Cy3:Alexa647 = 3:1 (6 mM:2 mM) to avoid the dually labeled product Cy3 bis-Alexa647. After 5 hours of incubation at 50 °C, we purified the Cy3-Alexa647 heterodimer by high performance liquid chromatography (1100 series, Agilent Technologies) using the C18 reverse phase column (RPC C2/C18 ST 4.6/100, GE Healthcare) with a polarity gradient by controlling the solvent ratio between acetonitrile (9017-03, J. T. Baker) and 5% v/v acetonitrile + 50 mM triethylammonium acetate (60-4110-57, Glen Research) solution. We used freeze-drying (FD8508, iLShinBioBase) to remove the buffer and obtain the heterodimer in a solid form.

### Characterization of the Cy3-Alexa647 heterodimer

The optical properties of the heterodimer were analyzed by UV/VIS absorption spectrometer (Lambda25, Perkin Elmer), fluorescence spectrometer (Quantamaster, Photon Technology International), quantum efficiency measurement system (QE-1200, Otsuka Electronics) and a home-built lifetime measurement system using a time-correlated single photon counter (TCSPC, SPC-150, Becker & Hickle) (see Supplementary Table S1, Fig. S4 and S5 online). In order to obtain the extinction coefficient, we measured the concentration of the heterodimer solution by using the fluorescence correlation spectroscopy (FLEX02-01D, Correlator.com), and calculated the extinction coefficient according to the Beer’s law since the total mass of the synthesized heterodimer was too small to be measured by our scale (<1 mg). The averaged efficiency of resonance energy transfer (RET) was calculated by dividing the integrated Alexa647 emission by the integrated Cy3-Aexa647 emission from the emission spectrum under 510-nm excitation.

### Labeling of secondary antibody with heterodimer

Immnunolabeling was carried out by reacting the Cy3-Alexa647 heterodimer with secondary antibodies (anti-mouse IgG produced in goat, F5262, Sigma Aldrich; anti-rabbit IgG produced in goat, A0545, Sigma Aldrich) in sodium borate buffer (0.1 M, pH = 8.5, 71999, Sigma Aldrich). The final concentrations of the reagents were roughly 200 μM and 4 μM for the heterodimer and secondary antibody, respectively. The amine groups of the secondary antibody react with the remaining NHS-ester functional group of the heterodimer. The reaction was kept on for more than 12 hours at 4 °C, after which the product was purified by a size-exclusive desalting column (PD-10, GE Healthcare) with phosphate buffered saline (PBS, 1×). We picked the fraction that shows three absorption peaks (protein, Cy3 and Alexa647) simultaneously in the UV/VIS absorption spectrum. By measuring the absorption and emission spectra, we confirmed that the optical properties of the heterodimer were well preserved after labeling (see Supplementary Fig. S5 online). We tested the activity of our heterodimer-labeled antibody by confirming the co-localization with commercially available FITC-labeled secondary antibody (F5262, Sigma Aldrich) (see Supplementary Fig. S6 online).

### Immunofluorescence labeling of cells

NIH3T3 (for nuclear pore complex) and primary Human-skin-fibroblast (for mitochondria and microtubule) cells were cultured on a microscope cover glass in a six-well plate (30006, SPL Lifesciences). We grew them in Dulbecco’s modified Eagle’s medium (LM001-79, Welgene) containing 10% v/v fetal bovine serum (16000-044, Gibco) and 1% v/v penicillin streptomycin (15140-122, Gibco) until their population was appropriate. We fixed the cells by using 4% of para-formaldehyde (P6148, Sigma Aldrich) with 0.1% glutaraldehyde (G5882, Sigma Aldrich) for 15 minutes and then treated with 0.1% sodium borohydride (213462, Sigma Aldrich) for 7 minutes to lower the background fluorescence. For the permeabilization and blocking processes, we incubated them at 4 °C for 30 minutes with a staining buffer containing 1% w/v bovine serum albumin (0332, Amresco) and 0.1% v/v triton X-100 (T8787, Sigma Aldrich) in PBS buffer. Subsequently, the primary antibody (Nup153: Ab24700, Abcam; Tom20: Ab56783, Abcam; Tubulin: T4026, Sigma Aldrich) was applied to the staining buffer with 1 μg/mL concentration, and the cells were incubated at room temperature for 1 hours. Prepared secondary antibody was diluted about 100 times using the staining buffer, applied to cells, and we incubated them in the same condition with the primary antibody. In each step, we washed the sample with 1× PBS buffer rigorously. For RESOLFT imaging, we exchanged the buffer to a solution with 10 mM Tris pH 8.0 (AM9855G, Ambion), 50 mM NaCl (S3014, Sigma Aldrich), an oxygen scavenging system made of 10% w/v β-D-glucose (G0047, TCI America), 50 μg/mL glucose oxidase (G2133, Sigma Aldrich), 10 μg/mL catalase (C30, Sigma Aldrich), and proper concentrations of primary thiols (βME: M6250, Sigma Aldrich; MEA: M6500, Sigma Aldrich; L-cys-ME: 30160, Fluka) and KI for further experiments. For the switching property of Atto532, the prepared cells were mounted with PVA (10981, Sigma Aldrich) instead of the imaging buffer. All the pump-probe experiments were performed on the heterodimer labeled microtubules in fixed cells.

## Additional Information

**How to cite this article**: Kwon, J. *et al.* RESOLFT nanoscopy with photoswitchable organic fluorophores. *Sci. Rep.* **5**, 17804; doi: 10.1038/srep17804 (2015).

## Supplementary Material

Supplementary Information
