# Supplementary material for: RESOLFT nanoscopy with photoswitchable organic fluorophores
Source: Sci Rep. 2015 Dec 7;5:17804. doi: 10.1038/srep17804 (PMC4671063; doi:10.1038/srep17804)
Supplement: Supplementary Information [file srep17804-s1.pdf]

*Supplementary Information*

# RESOLFT nanoscopy with photoswitchable organic fluorophores

Jiwoong Kwon,[1†] Jihee Hwang,[1†] Jaewan Park,[2] Gi Rim Han,[2]
Kyu Young Han[3,4] and Seong Keun Kim[1,2*]

[1]Department of Biophysics and Chemical Biology, Seoul National University, Seoul 151-747, Korea

[2]Department of Chemistry, Seoul National University, Seoul 151-747, Korea

[3]Howard Hughes Medical Institute, Urbana, Illinois 61801, USA

[4]Department of Physics and Center for the Physics of Living Cells, University of Illinois at Urbana-Champaign, Urbana, Illinois 61801, USA,

[†]These authors equally contributed to this work.

[*]Correspondence and requests for materials should be addressed to S.K.K. (seongkim@snu.ac.kr)

| Fluorophore | $\lambda_{max}$ (nm) | | $\varepsilon$ ($M^{-1}cm^{-1}$) | Q.Y. (%) | $F_D$ (%) | # of Cycles | Imaging Conditions ($nm/kW{*}cm^{-2}/\mu s$) | | | Resolution (nm) |
|---|---|---|---|---|---|---|---|---|---|---|
| | Abs. | Em. | | | | | On | Off | Excitation | |
| rsEGFP2[a] | 478 | 503 | 61,300 | 0.30 | ~10 | 6000 | 405/5/20 | 488/34/360 | 488/76/20 | 45 |
| C6-Ab670[b] | 458[c] | 505[c] | 54,000[c] | 0.78[c] | <10 | - | 543/40/ - | 375/0.2/ - | 415/0.02/ - | 48 |
| Rh-AA-DEA Particle[d] | 590 | 610 | 95,000 | 0.52 | ~6 | >60 | 671/ - / - | 375/ - / - | 543/ - / - | 80 ± 10 |
| Cy3-Alexa647[e] | 549<br>652 | 560<br>665 | 53,200<br>63,300 | 0.15[f] | <2 | >60 | 532/1.6/ 500 | 658/4.0/ 1500 | 633/2.1/ 500 | 74 ± 20 |

[a] From Grotjohann *et al.*, eLife, 1, e00248 (2012). [b] From Bossi *et al.*, New J. Phys. 8, 275 (2006) [c] Properties of Coumarin 6 from Reynords & Drexhage, Opt. Commun. 13, 222-225 (1975). [d] From Fölling *et al.*, Small, 4, 134-142 (2008). [e] This work. [f] Quantum yield of Alexa647 in the heterodimer. Symbol '-' marks unavailable data.

**Table S1** Comparison of rsEGFP2, the most-used RSFP, and organic dyes used in RESOLFT nanoscopy. Basic photophysical properties including absorption/emission maxima ($\lambda_{max}$-Abs./Em.), extinction coefficient ($\varepsilon$) and quantum yield (Q.Y.), as well as the photoswitching characteristics for RESOLFT nanoscopy, are presented. The extinction coefficient and quantum yield of Cy3-Alexa647 were measured in aqueous solution.

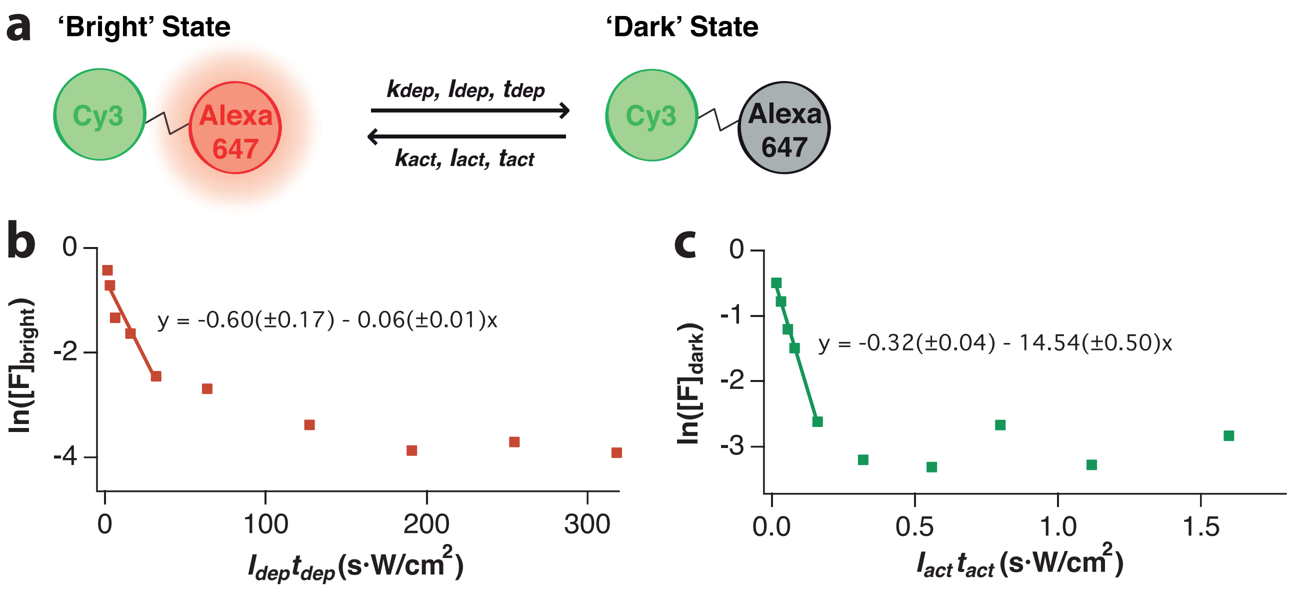


**Fig. S1** Determination of the kinetic rate constants ($k_{dep}$ and $k_{act}$) for switching processes from the pump-probe experiments. The 'bright' fluorophore turns into the 'dark' fluorophore under the illumination of depletion laser ($I_{dep}$) with the kinetic constant $k_{dep}$, while the 'dark' fluorophore is restored by activation laser ($I_{act}$) with the kinetic constant $k_{act}$ (Fig. S1a). By solving the following simple first-order coupled kinetic equations for this scheme (left), we derive the equations that describe the time-dependent population of each state (right):

$$\frac{d}{dt_{dep}}[F]_{dark} = k_{dep} I_{dep} [F]_{bright} \qquad [F]_{dark} = [F]_{total} - A \cdot \exp\left(-k_{dep} I_{dep} t_{dep}\right)$$

$$\frac{d}{dt_{act}}[F]_{bright} = k_{act} I_{act} [F]_{dark} \qquad [F]_{bright} = [F]_{total} - B \cdot \exp\left(-k_{act} I_{act} t_{act}\right)$$

Here, $t_{dep}$ and $t_{act}$ are the depletion and activation time, respectively, and $[F]_{dark}$ and $[F]_{bright}$ are the population of the 'dark' and 'bright' states. Their sum, $[F]_{total} = [F]_{dark} + [F]_{bright}$, is maintained constant by assuming that there is no photobleaching, which leads to the following semi-logarithmic linear relationships between fluorophore population and the product of fluorescence intensity and time, whose slopes represent the kinetic rate constants:

$$\ln\left([F]_{bright}\right) = \ln(A) - k_{dep}\left(I_{dep} t_{dep}\right)$$

$$\ln\left([F]_{dark}\right) = \ln(B) - k_{act}\left(I_{act} t_{act}\right)$$

Figure S1b and S1c show the semi-logarithmic plots from our experimental data, whose linear regions yield the kinetic rate constants of $k_{dep} = 0.06 \pm 0.01$ cm$^2$W$^{-1}$s$^{-1}$ and $k_{act} = 14.54 \pm 0.50$ cm$^2$W$^{-1}$s$^{-1}$, while their non-linear regions represent optical saturation at high photon flux.

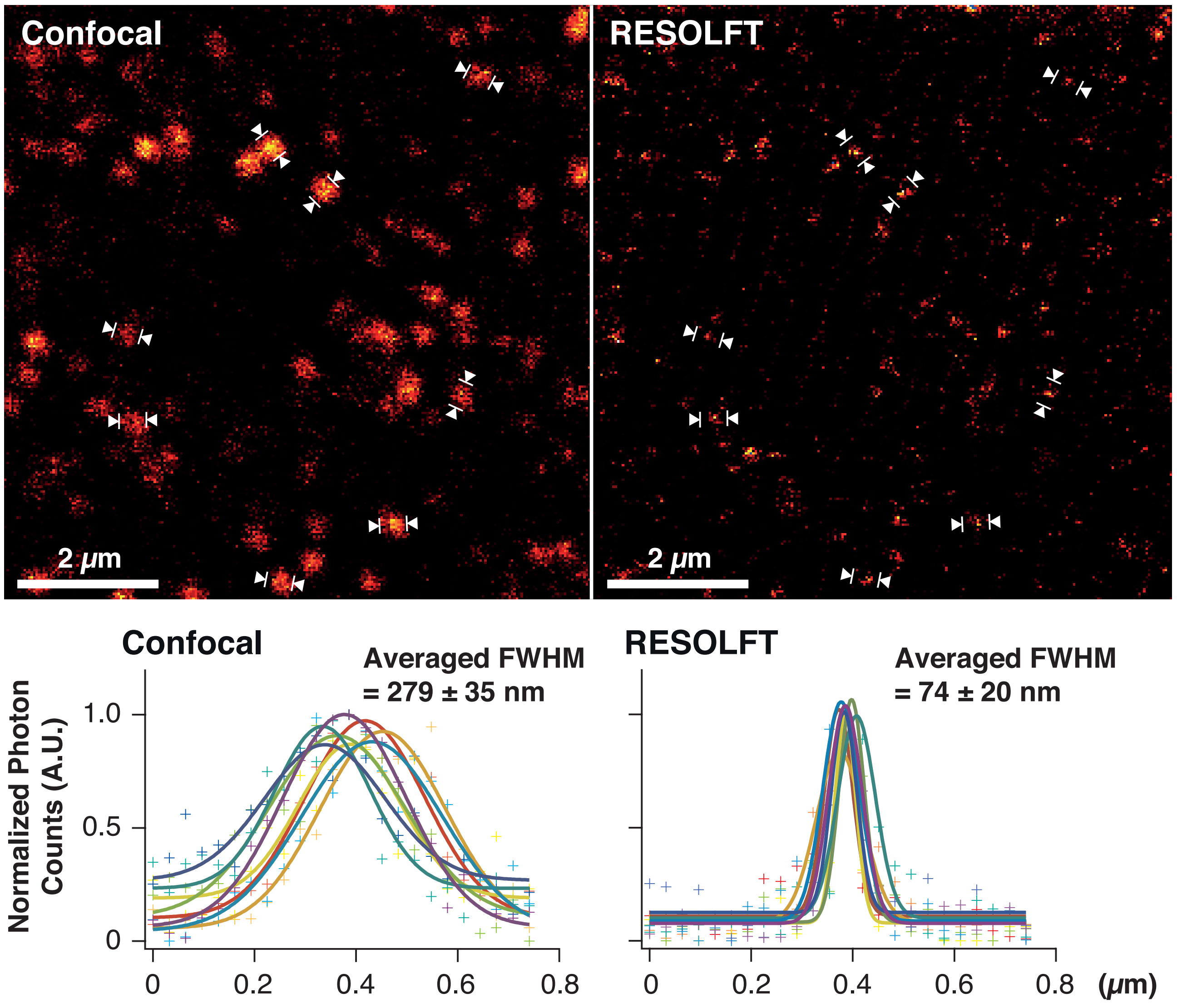


**Fig. S2** Fluorescence line profiles of eight randomly selected positions (white markers) from confocal and RESOLFT images. The raw profiles shown by '+' symbols and the corresponding Gaussian fits are color-coded.

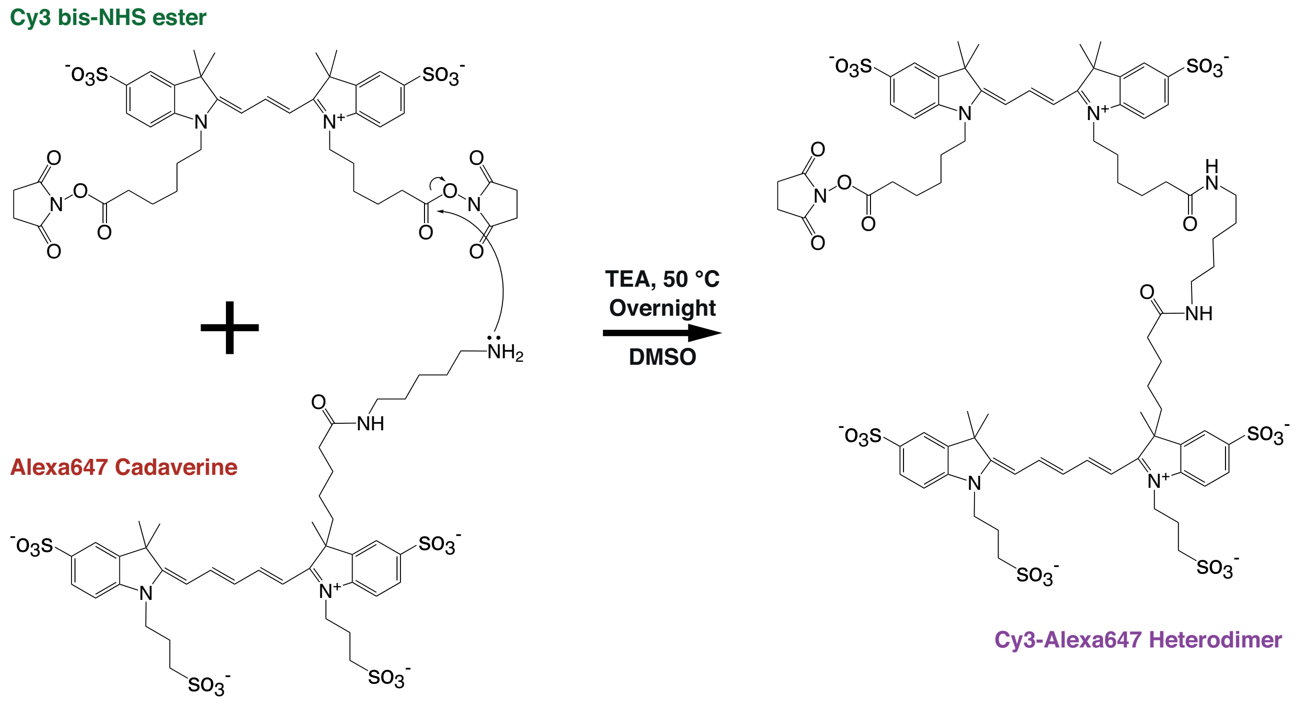


**Fig. S3** Scheme for the synthesis of covalently-linked Cy3-Alexa647 heterodimer through a click reaction between one of the NHS ester groups of Cy3 and the amine group of Alexa647. The overall reaction was carried out in anhydrous DMSO to prevent hydrolysis of the NHS ester group. The other remaining NHS ester group can be used for further labeling processes.

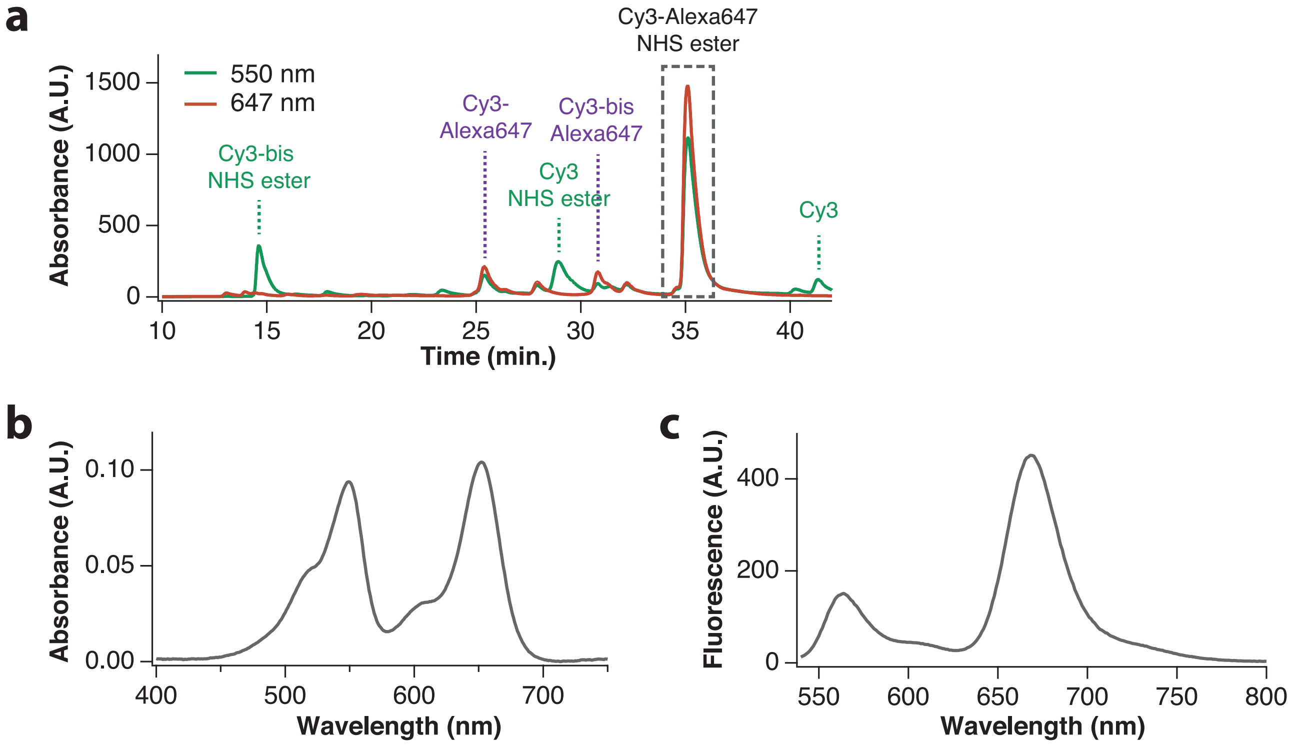


**Fig. S4** (a) HPLC data for purification of Cy3-Alexa647 heterodimer. The reactive heterodimer (in dashed-line box) was extracted at ~35 minutes and showed a strong dual-absorbance peak at 550 and 647 nm. We collected this fraction and confirmed its absorption and emission characteristics. Minor peaks were also assigned to individual fragmented fluorophores. (b) Absorption spectrum of Cy3-Alexa647 heterodimer. Both Cy3 and Alexa647 show their own absorption band but there is a slight change in the extinction coefficient due to the covalent bond formation. (c) Fluorescence emission spectrum of Cy3-Alexa647 heterodimer excited at 532 nm.

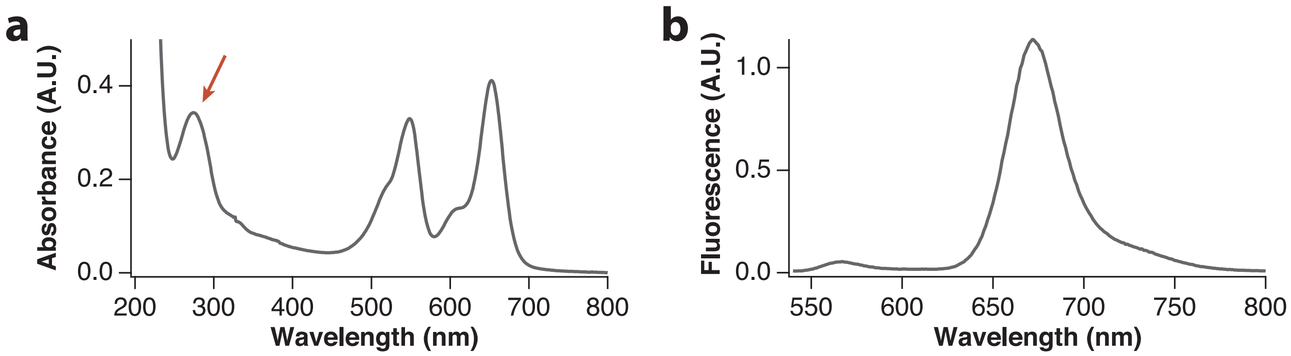


**Fig. S5** (a) Absorption spectrum of Cy3-Alexa647 labeled secondary antibody. The strong absorption bands in the UV region (< 300 nm) are due to the secondary antibody. The ratio between the three peaks at 280 nm (red arrow, antibody protein), 540 nm (Cy3), and 640 nm (Alexa647) was used to choose the correct fraction in the purification process. (b) Fluorescence emission spectrum of Cy3-Alexa647 labeled secondary antibody excited at 532 nm. The two emission bands are still visible although there is some enhancement in FRET efficiency due to the neighboring protein.

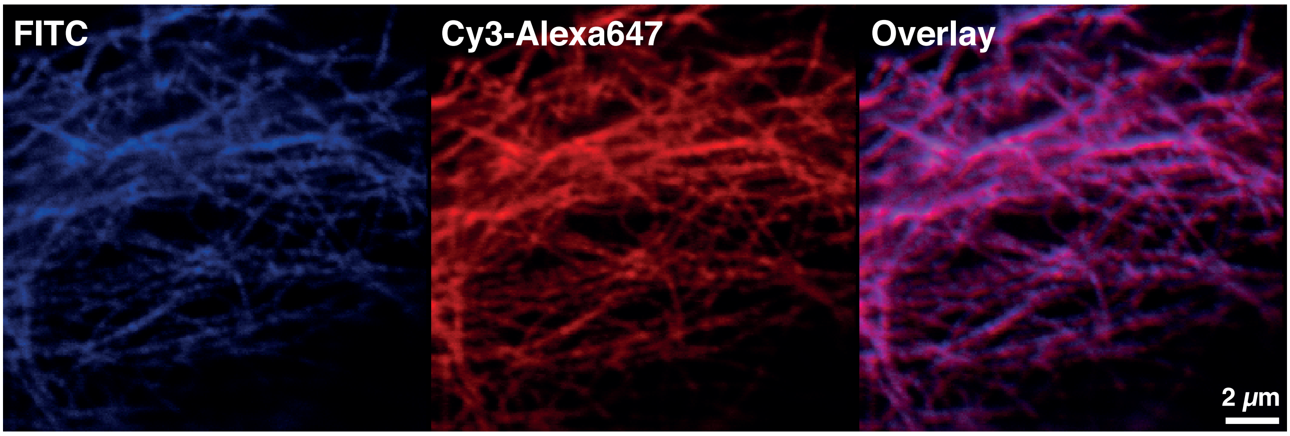


**Fig. S6** Verification of the activity of the Cy3-Alexa647 labeled secondary antibody by confocal microscopy. A commercially available secondary antibody, FITC (F5262, *Sigma Aldrich*), was used as the reference. The color pattern of the overlay image confirms that both signals from FITC and Cy3-Alexa647 are identically located, indicating that the labeling process of Cy3-Alexa647 on the secondary antibody does not affect the activity of the latter.
